# Human Antibodies that Recognize Novel Immunodominant Quaternary Epitopes on the HIV-1 Env Protein

**DOI:** 10.1371/journal.pone.0158861

**Published:** 2016-07-13

**Authors:** Mark D. Hicar, Xuemin Chen, Chidananda Sulli, Trevor Barnes, Jason Goodman, Hakimuddin Sojar, Bryan Briney, Jordan Willis, Valentine U. Chukwuma, Spyros A. Kalams, Benjamin J. Doranz, Paul Spearman, James E. Crowe

**Affiliations:** 1 Departments of Pediatrics, University at Buffalo, Buffalo, New York, United States of America; 2 Departments of Microbiology and Immunology, University at Buffalo, Buffalo, New York, United States of America; 3 Departments of Pediatrics, Microbiology and Immunology, Emory University School of Medicine, Atlanta, Georgia, United States of America; 4 Integral Molecular, Inc., Philadelphia, Pennsylvania, United States of America; 5 Departments of Pathology, Microbiology and Immunology, Vanderbilt University Medical Center, Nashville, Tennessee, United States of America; 6 The Program in Chemical Biology, Vanderbilt University Medical Center, Nashville, Tennessee, United States of America; 7 The Department of Medicine, Vanderbilt University Medical Center, Nashville, Tennessee, United States of America; 8 Children’s Healthcare of Atlanta, Atlanta, Georgia, United States of America; 9 The Vanderbilt Vaccine Center, Vanderbilt University Medical Center, Nashville, Tennessee, United States of America; Simon Fraser University, CANADA

## Abstract

Numerous broadly neutralizing antibodies (Abs) target epitopes that are formed or enhanced during mature HIV envelope formation (*i*.*e*. quaternary epitopes). Generally, it is thought that Env epitopes that induce broadly neutralizing Abs are difficult to access and poorly immunogenic because of the characteristic oligomerization, conformational flexibility, sequence diversity and extensive glycosylation of Env protein. To enhance for isolation of quaternary epitope-targeting Abs (QtAbs), we previously used HIV virus-like particles (VLPs) to bind B cells from long-term non-progressor subjects to identify a panel of monoclonal Abs. When expressed as recombinant full-length Abs, a subset of these novel Abs exhibited the binding profiles of QtAbs, as they either failed to bind to monomeric Env protein or showed much higher affinity for Env trimers and VLPs. These QtAbs represented a significant proportion of the B-cell response identified with VLPs. The Ab genes of these clones were highly mutated, but they did not neutralize common HIV strains. We sought to further define the epitopes targeted by these QtAbs. Competition-binding and mapping studies revealed these Abs targeted four separate epitopes; they also failed to compete for binding by Abs to known major neutralizing epitopes. Detailed epitope mapping studies revealed that two of the four epitopes were located in the gp41 subunit of Env. These QtAbs bound pre-fusion forms of antigen and showed differential binding kinetics depending on whether oligomers were produced as recombinant gp140 trimers or as full-length Env incorporated into VLPs. Antigenic regions within gp41 present unexpectedly diverse structural epitopes, including these QtAb epitopes, which may be targeted by the naturally occurring Ab response to HIV infection.

## Introduction

An increasing number of broadly neutralizing monoclonal Abs (mAbs) against HIV have been identified in recent years and have illuminated new neutralizing epitopes on the envelope (Env) glycoprotein complex. It remains unclear why the HIV virion does not induce broadly neutralizing Abs earlier in infection, or in a higher percentage of infected individuals. Generally, it is thought that the broadly neutralizing epitopes may be relatively weakly immunogenic and structurally difficult to access [1]. It has been postulated that targeting of conformational epitopes on mature Env trimers may be necessary to neutralize HIV. Conformational epitopes within mature Env can be found within a single protomer, and distant patches of amino acid sequences that are brought together by protein folding. In contrast, quaternary epitopes are conformational epitopes whose structure depends upon the arrangement of multiple protomers, or is enhanced by them, or may depend on the arrangement of multiple protomers (*i*.*e*., quaternary epitopes). A quaternary epitope may be located in a single protein of a multimeric complex, or it may span multiple protomers, being formed *de novo* by their interaction. Previous studies that identified the broadly neutralizing mAbs PG9 and PG16, which preferentially recognize novel quaternary epitopes, also showed that the overwhelming majority of the neutralizing anti-HIV supernatants from B-cell cultures did not bind to monomeric gp120 or gp41 proteins [2]. However, the induction of individual broadly neutralizing Abs appears to be relatively rare and often occurs late in infection [3, 4].

Designing the ideal antigen for an HIV vaccine is a current challenge in the field. Monomeric Envs generally induce neutralizing Ab responses of limited breadth and depth [5, 6]. Scheid *et al*. cloned collections of anti-HIV Abs using a flow cytometric sorting technique and selection of single B cells with recombinant gp140 trimers comprising gp120 and the ectodomain of gp41 trimerized with the T4-phage fibritin trimerization motif -(termed gp140 foldons) [7]. Many of the variable genes encoding these Abs possess a high level of somatic mutation. Interestingly, none of these Abs were specific for trimeric Env since they also recognized monomeric recombinant gp120. When a similar gp140 foldon construct was used as vaccine in rhesus macaque challenge studies, protection against heterologous rectal simian-HIV (SHIV) challenge was only modest [8]. Recombinant Env proteins generally exhibit a poor ability to induce a broad neutralizing Ab response. This finding may be related to difficulty in recapitulating conformational, and specifically quaternary, epitopes in recombinant Env proteins.

We previously developed a pseudovirion- or virus-like particle (VLP)-based platform for antigen presentation of naturally cleaved Env trimer [9]. These VLPs present the epitopes for classic broadly neutralizing HIV mAbs and responded to CD4 binding by increasing access to CD4-induced epitopes [10]. To study quaternary epitope-targeting Abs (QtAbs), we used these VLPs in fluorescence-activated flow-cytometric cell sorting experiments to isolate mAbs including a neutralizing human mAb to a CD4-induced quaternary epitope [10, 11]. We also identified a panel of novel Abs that possess a large number of somatic mutations, but do not exhibit broad or potent Ab-dependent cell-mediated virus inhibition or HIV neutralization [12]. Env trimers and VLPs, rather than monomeric Env, are the preferred antigen targets of these Abs, which is consistent with their being QtAbs.

In this study, to further the characterization of these QtAbs, we sought to define the epitopes targeted by members of this panel of QtAbs. Competition-binding studies showed these QtAbs targeted four separate quaternary epitopes on the HIV virion (designated here Competition Groups [CGs] A-D). These QtAbs did not bind monomeric gp120 or gp41 ectodomain readily and did not bind linear epitopes in binding screens with Env peptides. Surprsingly, they did not compete for binding to trimeric Env with a panel of known broadly neutralizing mAbs that are QtAbs. Alanine-scanning mutagenesis studies were used to map two of these epitope CGs (B and C). One CG (B) targeted the gp41 immunodominant 1 region known to undergo dynamic changes during fusion, and the second CG (C) targeted a discontinuous epitope that had not been previously described. These immunogenic epitopes should be considered during structure-based vaccine design of optimal HIV Env immunogens.

## Materials and Methods

### Full-length Ab expression

We previously reported the isolation from several long-term non-progressor subjects of a large panel of human mAbs to HIV from peripheral blood B cells labeled with VLPs [10–12]. The cells were obtained from a de-identified sample repository. The prior research involving human participants to obtain the samples for the repository was approved by the Vanderbilt Institutional Review Board (IRB), and all clinical investigation was conducted according to the principles expressed in the Declaration of Helsinki. Written informed consent was obtained from all participants. Ab heavy and light chain variable gene segments isolated from individual B cells from HIV- infected long-term non-progressors were cloned into a full-length human IgG1 expression vector as previously described [10]. Recombinant IgG1 Abs were prepared from transient transfection and purified on Protein G columns as described previously [10].

### Biotinylated Ab competition assays

Abs first were equilibrated in carbonate buffer (pH 9.3) using 50-kDa cutoff Amicon Ultra-4 centrifugal filter units (Millipore Corp., Billerica, MA) per the manufacturer’s instructions. Ab concentrations were determined using a Nanodrop spectrophotometer (Thermo Scientific). Sulfosuccinimidobiotin (Sigma-Aldrich, St. Louis, MO) was added at a 30-fold molar excess over the concentration of Ab (approximately 90 μg per mg of Ab). This solution was mixed and incubated for 30 minutes with gentle shaking at room temperature. Trizma-HCl (1M, pH 7.5) was added up to 10% by volume to bind residual biotin molecules. The biotinylated Ab was separated from residual biotin complexes and equilibrated to PBS (pH 7.4) using a G-25 Sephadex Nap-5 column (GE Healthcare, Piscataway NJ) per the manufacturer’s protocol. Biotinylated Abs were competed with the panel of known unlabeled HIV-specific Abs, as listed in the results section. Gp140 trimers or VLPs in PBS were added to the wells of Immulon II HB 96-well microplates (Dynex Technologies, Inc., Chantilly, VA) and stored at 4°C overnight. BaL gp140 trimers, more specifically gp140 foldons, were produced with transient transfection in HEK 293 FreeStyle cells (Invitrogen, Carlsbad, CA). The BaL foldon expression construct consisted of BaL gp140, a T4-phage fibritin trimerization domain (*i*.*e*., foldon domain) [13], and a his-tag sequence prior to the stop codon. Synthesis of this construct was ordered from GenScript (Piscataway, NJ), cloned in-frame in the pcDNA 3.1 expression vector. These constructs have a retained furin cleavage site (REKR). Supernatants were filtered initially with a 0.2 micron filter and further purified using using an Amicon Ultra-50, PLHK Ultracel-PL membrane with a 150-kDa cutoff (Millipore Corp., Billerica, MA). Native and denatured polyacrylamide gel electrophoresis was used in tandem to check for purity and resolution of the trimer prior to use. The SF162 gp140 trimer was obtained from NIH AIDS Reagent Repository, and also retains its furin cleavage site [14, 15]. For the gp140 trimer assays, plates were coated with 100 μL of gp140 trimer at 10 ng/mL at 4°C overnight. VLPs were produced similarly to our previously described work with an HIV VLP ELISA [10, 11]. The incorporation of Env can vary between VLP preparations. To control for this variation, we used control Abs to optimize concentrations of VLPs used in each ELISA. After creation of VLPs, a small aliquot of each batch was removed, diluted in various concentrations, and used as the capture antigen in a VLP ELISA. Binding of Abs 2G12 and 2F5 were used to choose the optimal (best dynamic range with lowest background) VLP dilution for each individual batch. Quantification of antigen on the surface of these types of VLPs is detailed in previous work [9]. From that work, we estimated that 300–500 picograms of Env incorporated into VLP was used per well on the ELISA plates. VLP was added in 100 μL of PBS to each well. After overnight incubation, coating solution was removed and the plates were blocked with PBS containing 10% FBS for 2 hours at room temperature. EC_50_ values of Abs were predetermined against test antigen by using two-fold dilutions ranging from μg/mL to picogram/mL of the test Ab. Non-linear regression using Prism Software (GraphPad, La Jolla, CA) was performed on the binding results to estimate each EC_50_. Results shown are from use of a normalized unlabeled-to-labeled Ab concentrations ratio of 2:1, based EC_50_. For example, if the EC_50_ of an Ab was 20 ng/mL, then the unlabeled Ab was plated at 40 ng/mL against the labeled Ab concentration of 20 ng/mL. After blocking, Abs were mixed in diluent (PBS containing 7.5% FBS) and added to microtiter wells at 2:1 unlabeled/labeled ratio (normalized to EC_50_), and incubated for 1 h at room temperature. Plates were washed and incubated with a 1:5,000 mix of streptavidin-horseradish peroxidase (HRP) in diluent (Southern Biotech, Birmingham, Al) for 1 h at room temperature. Plates were washed three times with PBS, then incubated with 100 μL per well of 1-Step Ultra TMB-ELISA Substrate Solution (Thermo Fisher, Grand Island, NY). 100 μL of 2 N sulfuric acid was used to stop the reaction. Plates then were read with a μQuant microplate spectrophotometer (BioTek, Winooski VT) at 450 nanometers.

### Peptide binding assays

Group M consensus peptides (obtained through the NIH AIDS Reagent Program, Division of AIDS, NIAID, NIH: HIV-1 Consensus Group M Env Peptide Set Cat# 9487) were dissolved in 10% (v/v) DMSO in PBS. Peptides were diluted further in PBS, and microwells were incubated with 50 μg per well of peptide overnight at 4C on a rocking platform. Wells were washed in PBS three times and blocked with 10% (w/v) BSA in PBS. Test Abs (100 ng/mL) were added to the wells and incubated at 37°C for 1 hour. After washing, wells were incubated with secondary HRP-conjugated goat anti-human IgG (H+L) (Southern Biotech, Birmingham, AL) diluted 1:1,000 in PBS for 1 hour at room temperature. After washing, 1-Step Ultra TMB-ELISA Substrate Solution (Thermo Fisher, Grand Island, NY) was added and color development was halted with 2N sulfuric acid similar to the assays mentioned above. Optical densities were read at 450 nm absorbance on a μQuant microplate spectrophotometer (BioTek, Winooski VT), and data were analyzed with Prism Software (GraphPad, La Jolla, CA).

### Alanine scanning mutagenesis

A shotgun mutagenesis mutation library was created for HIV-1 (strain KNH1144, GenBank #JQ715384), as previously described [16]. Briefly, a parental plasmid expressing codon-optimized gp160 Env (truncated at residue 708) was used as a template to make a library of alanine substitutions across gp160 targeted residues 30–708. Each residue was mutated to alanine; alanine residues were mutated to serine. In total, 678 Env mutants were generated (99.9% coverage), sequence confirmed, and plasmids expressing each mutant were arrayed into 384-well plates (one mutant plasmid per well). Each mutation array plate contained mutants plus eight positive wells (containing cell lines with native Env expression) and four negative control wells (containing the cell line only). Each mutant was used to transfect human HEK-293T cells and expression proceeded over 22 h with incubation in 5% CO_2_ and at 37°C. Cells were fixed in 4% (v/v) paraformaldehyde (Electron Microscopy Sciences, Hatfield, PA) in PBS plus calcium and magnesium (PBS++). Cells were stained with optimized concentrations (0.1–2.0 μg/mL) of purified mAbs diluted in PBS containing 10% normal goat serum (NGS, Sigma-Aldrich, St. Louis, MO). The optimized primary Ab concentration was determined separately for each Ab by titrating Ab immunofluorescence against the parental wild-type gp160 to ensure that signals were within the linear range of detection, and that the difference between signal and background was at least 5-fold. Bound Abs were detected using 3.75 μg/mL AlexaFluor488-conjugated goat anti-human IgG (Jackson ImmunoResearch Laboratories, Pike West Grove, PA) diluted 1:1,000 in PBS containing 10% NGS, after which wells were washed three times with PBS. Mean cellular fluorescence was detected using the Intellicyt High-Throughput Flow Cytometer (HTFC, Intellicyt, Albuquerque, NM). Ab reactivities against each mutant gp160 clone were calculated relative to wild-type Env reactivity by subtracting the signal from mock-transfected controls, then normalizing to the signal from wild-type Env-transfected controls. Residues required for mAb binding were identified as critical to the mAb epitope if reactivity of the test mAb was lost (<20–35% of wild-type), but reactivity of control Abs (>70%) was retained. This counter-screen strategy enabled the exclusion of gp160 mutants that were locally misfolded or that had an expression defect [16]. Critical residues required for Ab binding were visualized on the SIV gp41 post-fusion structure (PDB ID 2EQ) [17] and BG505 SOSIP.664 HIV-1 Env trimer (PDB ID 4ZMJ) [18].

## Results

Previously, we identified a panel of highly mutated non-neutralizing mAbs from a cohort of HIV-infected long-term non-progressor subjects [12]. A number of these mAbs preferentially bound BaL-strain Env in trimeric forms, and not monomeric proteins, consistent with the functional pattern of binding of QtAbs. These QtAbs and mAbs from the same study with significant sequence homology represented a significant proportion of the B-cell response identified with VLPs. Because these mAbs represent a dominant population of the Abs identified by binding to Env presented on VLPs, we sought to further determine the epitopes targeted by these Abs. Here, we fully characterized a collection of ten Abs from one subject (10076) that represented a total of eight clonal groups of mAbs previously assigned by sequence similarity [12]. The expressed Ab genes and sequence information for these ten recombinant mAbs are shown in detail in S1 Table.

**Fig 1:**
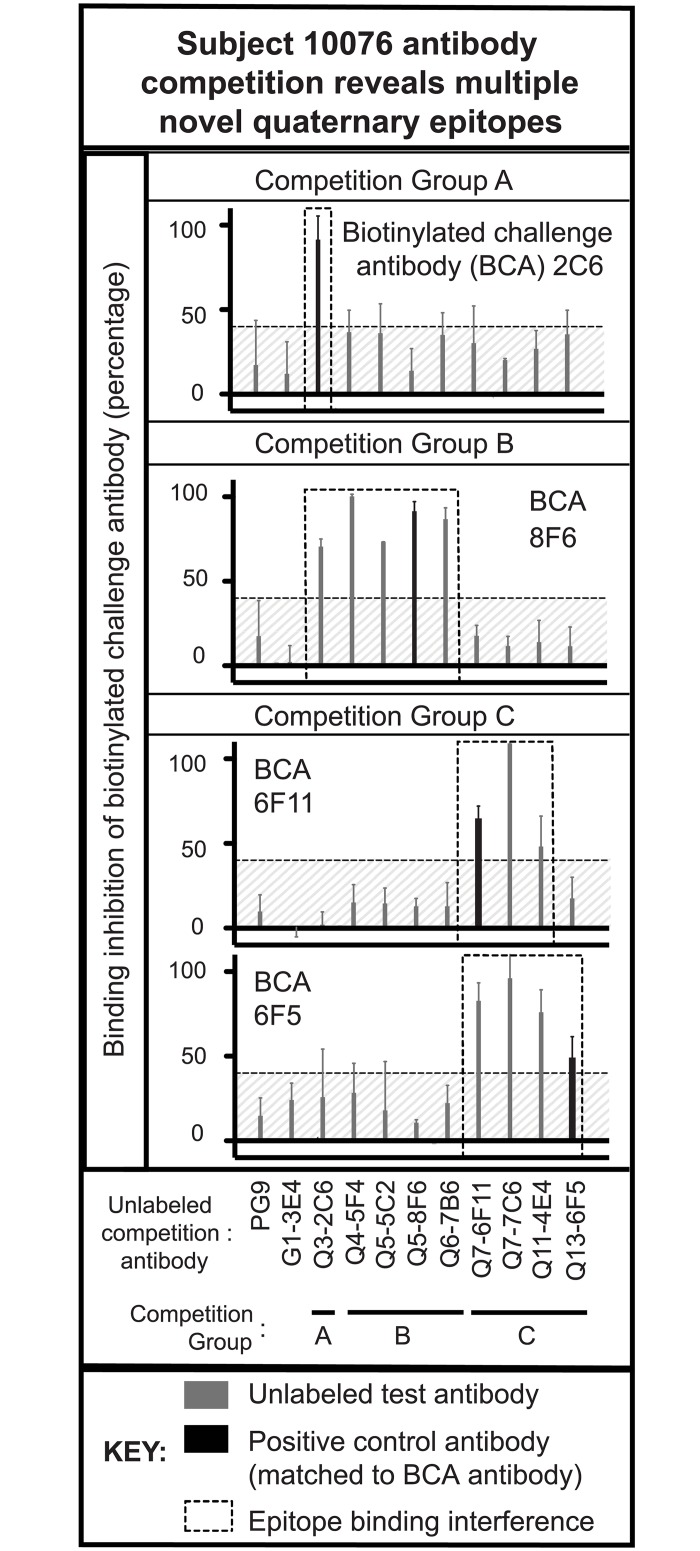
QtAb competition ELISA against BaL gp140 trimers revealed multiple novel quaternary epitopes. Each frame represents competitive binding against a different biotinylated competitor Ab (BCA) (using QtAbs 2C6, 8F6, 6F11, or 6F5). Unlabeled Ab to BCA ratios of 2:1 (based on EC_50_ values) are shown. Competition was confirmed by control Ab inhibition (the unlabeled Ab was same Ab as the BCA) shown in thick black bars for each frame. Competitive inhibition of BCA binding of >40% (mean value irrespective of standard deviation), approximately twice the background % of controls (outside of the diagonally shaded areas beneath the dashed line), was considered positive. Assays were performed in duplicate and repeated twice, with consistent inter-assay results. The Abs within each dashed box represents those Abs forming a CG (A, B or C). The negative control, mAb PG9, targets a well-characterized neutralizing quaternary epitope; as an unlabeled competitor, it did not show any competition. Another unlabeled competitor, mAb G1-3E4, is a non-QtAb control Ab from the same study.

To further define the epitopes recognized by these QtAbs, we developed a competition-binding ELISA using biotinylated forms of recombinant full-length QtAbs, whose variable regions are encoded by expressed Ab genes of VLP-Env-specific B cells (biotinylated challenge Ab [BCA], Fig 1). Due to the disparate binding affinities, Ab concentrations were normalized to EC_50_s (Table 1) as detailed in the methods. The previously identified QtAb, PG9, which recognizes a conformational epitope in the gp120 V1/V2 region [2] that was first identified by mAb 2909 [19], did not compete for binding (shown in Fig 1). PG9 also required the use of concentrations in the microgram per mL amount to achieve normalized EC_50_ ratios of 2:1 for these assays. Fig 1 shows the results using SF162-Env gp140 trimers, (gift of L. Stamatatos), and four representative QtAb BCAs 76-Q3-2C6, 76-Q5-8F6, 76-Q11-6F11 or 76-Q13-6F5). For convenience, full clone names were shortened to three-letter unique designators throughout the rest of this manuscript (*i*.*e*. 76-Q3-2C6 is designated as 2C6). An unlabeled Ab to BCA ratio of 2:1, normalized to EC_50_ values, was used with readout as reduction of streptavidin-HRP signal, reflecting inhibition of binding. A normalized EC_50_ 2:1 ratio of unlabeled Ab to BCA ratio showed reduction of streptavidin-HRP signal when competed with the same non-labeled Ab (thick black column) as an internal control. Unlabeled Abs competing against the same BCA in this assay were considered part of that BCA epitope CG (columns outlined with dashed boxes). Abs whose average (irrespective of standard deviation) competition was over 40%, which was twice the average level of nonspecific competition observed in controls, were defined as competing Abs (Fig 1, shaded hatched portion). The results from testing the QtAbs segregated the Abs into four CGs, designated CGs A-D).

**Table 1:** Epitope competition group (CG) assignment, binding kinetics and preference of quaternary-epitope targeting Abs for binding to Env in VLPs gp140 trimers.

Epitope Competition groups (CGs)^a^	Subj.10076 clonal group^b^	mAb	gp41^c^	VLP binding preferred	HIV clade B virus trimeric Env protein antigens					
					VLP^c^ BaL strain		gp140 trimers			
							BaL strain		SF162 strain	
					EC_50_ [ng/mL] (95% CI)	slope	EC_50_ [ng/mL] (95% CI)	slope	EC_50_ [ng/mL] (95% CI)	slope
A	Q3	2C6	>	Yes	0.87 (0.75–1.0)	1.9	10.0 (8.3–12.2)	1.8	45.3 (39.3–52.3)	1.5
B	Q4	5F4	500–1,000	Yes	2.0 (1.8–2.2)	1.6 ^d^	24.4 (19.6–30.4)	1.0 ^d^	> ^e^	nd ^f^
	Q5	5C2	315.9 (171.2–583.0)	Yes	2.5 (2.3–2.8)	1.8 ^d^	44.5 (30.1–64.7)	0.8 ^d^	>	nd
		8F6	nd	Yes	2.6 (2.1–3.2)	1.2	129.0 (90.0–185)	0.9	>	nd
	Q6	7B6	nd	Yes	2.6 (2.4–2.8)	1.3	66.4 (50.0–87.9)	0.9	>	nd
C	Q7	6F11	nd	No	10.8 (8.7–13.3)	1.1 ^g^	13.2 (12.0–14.5)	1.7 ^e^	7.2 (6.5–7.9)	1.4 ^g^
		7C6	9.2 (6.5–13.1)	No	4.4 (3.6–5.5)	1.2 ^g^	5.7 (5.1–6.3)	1.6 ^e^	8.6 (7.6–9.7)	1.5 ^g^
	Q11	4E4	nd	Yes	11.2 (10.1–14.1)	1.3	33.1 (29.0–37.9)	1.1	44.5 (40.3–49.3)	1.4
	Q13	6F5	500–1,000	Yes	20.6 (16.0–26.0)	1.5 ^d^	631 (100–3900)	0.7 ^d^	>	nd
D	Q14	8B10	>	Yes	1.8 (1.4–2.3)	2.2	>	nd	>	nd

^a^ Competition Group (CG), determined by competition against binding of biotinylated Abs (see Fig 1).

^b^ Subject 10076 clones with quaternary (Q) epitope binding patterns were grouped by common genetic elements [12].

^c^ Gp41 and BaL strain VLP EC_50_ data were previously published, Table 3 of reference [12].

^d^ Indicates cooperative binding preference for VLP compared to gp140 trimers.

^e^ > Indicates EC_50_ was >1,000 ng/mL.

^f^ nd–Indicates ‘not determined’.

^g^ Indicates cooperative binding preference for gp140 trimers compared to VLP.

CGs A, B and C were defined by competition with unlabeled QtAbs for binding to BaL Env trimers, as shown in Fig 1. Competition-binding ELISAs showed similar results when performed using either VLPs (data not shown) or gp140 trimers from strain BaL for CGs A-C. The CG A QtAb 2C6 is interesting, because no other Ab interfered with its binding. However, it blocked binding of the CG B mAb 8F6. These results probably reflect the stronger affinity for antigen that 2C6 has relative to 8F6. Similarly, the CG C QtAbs 6F5 and 6F11 differ by two-fold in their EC_50_ values; the stronger binder, 6F11, inhibited BCA 6F5 strongly, whereas 6F5, the weaker binder of the two, only weakly inhibited BCA 6F11. QtAb 8B10 did not bind trimers readily (see below and Table 1), but using VLPs and the CG A-C BCAs, competition was not detected (data not shown). Hence, QtAb 8B10 was classified as representing a fourth competition-binding group, designated CG D. Representative BCAs of CGs A-C did not reveal competition between our QtAbs and representative mAbs that target gp120 (2G12, VRC01/03, B12, 447-52D), gp41 (2F5, 4E10, 50–69), CD4-induced Env epitopes (48d, 17b, F825), or conformational epitope targeting Abs (HJ16, F105 or A32) (data not shown). MAbs specific to immunodominant I and II regions are shown below in their respective specific mapping sections. These assays were performed with labeled Abs at concentrations corresponding to their EC_50_ values, and unlabeled Abs at concentrations twice their EC_50_ This design resulted in the use of mAbs in concentrations of 200–1,000 ng/mL in competition against the BCA at a concentration of 20 ng/mL.

We had determined previously the binding affinities of these mAbs against VLPs and showed that a subset of these mAbs bind poorly, if at all, to monomeric BaL gp120 or gp41 of strain III B, consistent with the designation QtAb [12]. To confirm that these QtAbs did not target linear epitopes, we performed Env-peptide-based epitope mapping of representative Abs from CGs A-C. The group M gp160 consensus peptides obtained from the NIH AIDS Reagents Program (Cat# 9487, individual Cat #s 8974–9184) are 15-mers with 11 amino-acid overlapping sequences. Binding by mAb 2C6 (CG A), mAb 7B6 (CG B), mAb 7C6 (CG C), control mAbs 2F5 (gp41 membrane proximal region) or 2G12 (gp120 glycosylation-dependent) was compared to account for non-specific binding., Identification of an epitope was considered reliable if the mAb bound specifically to two or more adjacent peptides. The utility of this restrictive screening method was confirmed by binding results of the control mAb 2F5 that bound specifically to three overlapping peptides (NIH AIDS Reagents Program Cat# 9136–9138; HXB2 gp160 reference amino acids 653–675). These three peptides contain the known 2F5 MPER epitope ELDKWA (HXB2 gp160 reference amino acids 662–667). For the peptides overlapping gp41, no adjacent peptides showed specific binding to any of the representative CGs A-C Abs, suggesting their epitopes are not present on short linear peptides (data not shown). Peptides that did show nonspecific binding (one control and multiple test Abs bound) were Cat #s 9112, 9113, 9120, 9123 and 9139. Notably, 2C6 did bind specifically to one peptide, but both adjacent peptide showed no binding, and this is currently being investigated.

**Fig 2:**
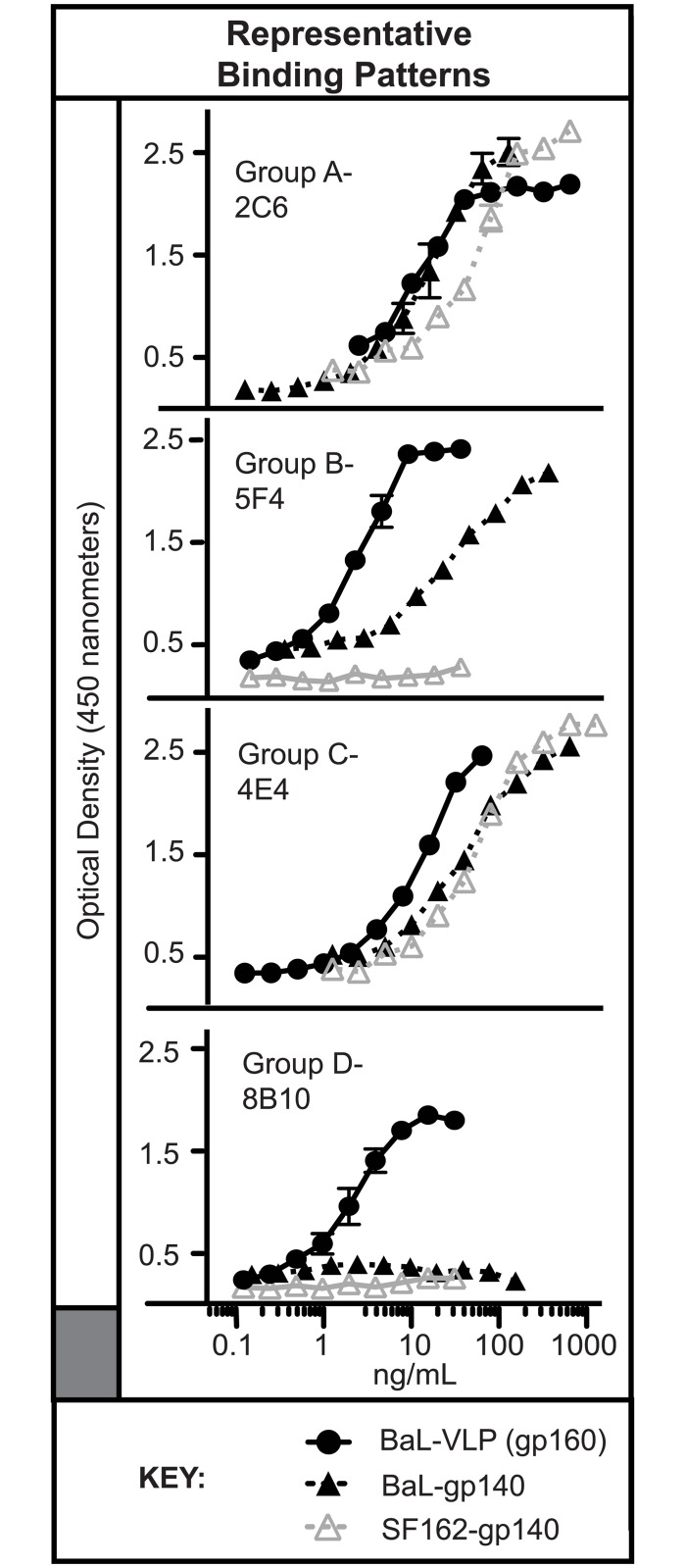
Quaternary epitope-targeting Abs variably bind gp140 trimers in a strain-dependent manner. Depending on the QtAb, variability was observed in binding BaL strain Env protein incorporated in VLPs (filled circle) versus gp140 trimers of the same strain (Clade B BaL, filled triangle) or of a different clade B strain (SF162, open triangle). See Table 1 for corresponding binding data.

To explore further the targeting of different Env forms, we compared binding of the 10 QtAbs to VLPs and gp140 trimers. We generated titration ELISA curves for these QtAbs against BaL strain VLPs or gp140 trimeric Env antigens from each of two strains, BaL and SF162 (examples are shown in Fig 2, with full results in Table 1). Most of the QtAbs exhibited a significant preference for the VLP form of Env. The most striking example of this pattern was the CG D Ab 8B10, which bound to BaL gp160 incorporated into VLPs, but not to BaL gp140 trimers (bottom panel of Fig 2). Some Abs exhibited slight preferential binding to the VLP antigen (for example, mAb 2C6). This diversity in patterns of recognition among these Ab CGs suggests that avidity alone did not drive the differential binding of Abs to VLP versus trimers. To assess if additional epitopes were influenced by VLP presentation, we analyzed the binding preference of the V3 loop-specific non-QtAb 447-52D. In contrast to the QtAbs, mAb 447-52D exhibited increased affinity for binding to Env trimers compared to VLPs (EC_50_ values 138 and 440 ng/mL, respectively). Intriguingly, the different antigens also revealed potential differences in cooperative binding of the QtAbs (revealed by difference in the slope of the binding curves, or Hill slope) [20]. Standard binding has a Hill slope of 1.0, and values greater than 1.0 imply some cooperative effect or avidity effect. This Hill slope varied between binding to VLP or gp140 trimer suggesting that recognition of an epitope may be affected by the format of presentation. Generally, CG B QtAbs were much more homogenous in their binding patterns than CG C QtAbs, however a subset of CG C (QtAbs 5F4 and 5C2) showed more cooperative binding when targeting their epitopes on VLPs versus gp140 trimers (Table 1).

**Fig 3:**
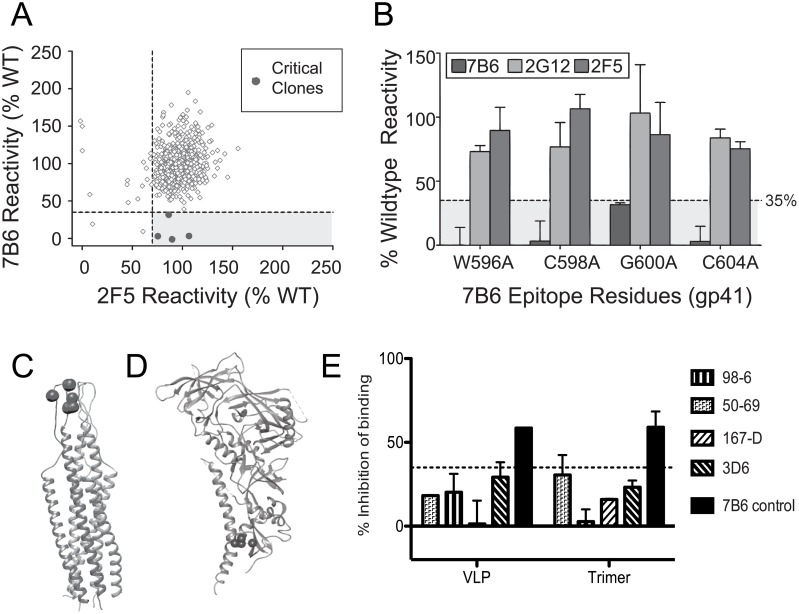
Identification of critical binding residues in the epitope of QtAb 7B6 by shotgun mutagenesis epitope mapping. (A) Binding of QtAb to mutant Env relative to wild-type HIV gp160 is plotted for each amino acid mutant for the test QtAb, as shown in this example of mAb 7B6 in comparison to a control mAb (2F5). Clones with reactivity <35% for the test mAb but with >70% reactivity for the control mAb were identified as critical for test mAb binding. (B) Alanine mutation of 4 individual residues significantly reduced 7B6 binding (dark grey bars), but did not impair binding of control Abs (2G12 or 2F5, shown in light grey or mid grey). Bars represent the mean and range of two replicates. (C) Structural analysis of the critical residues required for 7B6 binding. Critical residues are shown on one chain of the gp41 post-fusion trimer (PDB 2EZQ) and (D) the BG505 SOSIP.664 HIV-1 Env trimer (PDB 4ZMJ). (E) Biotinylated competition Ab 7B6 was competed against known gp41 mAbs, including Cluster I mAb 3D6 (thick diagonal). The experiment contained duplicate wells and was repeated twice. Competition of >40% inhibition of binding of the BCA, approximately twice the background % of controls (over hatched line), was considered a positive result and only shown with the control 7B6 unlabeled Ab (filled bar).

**Fig 4:**
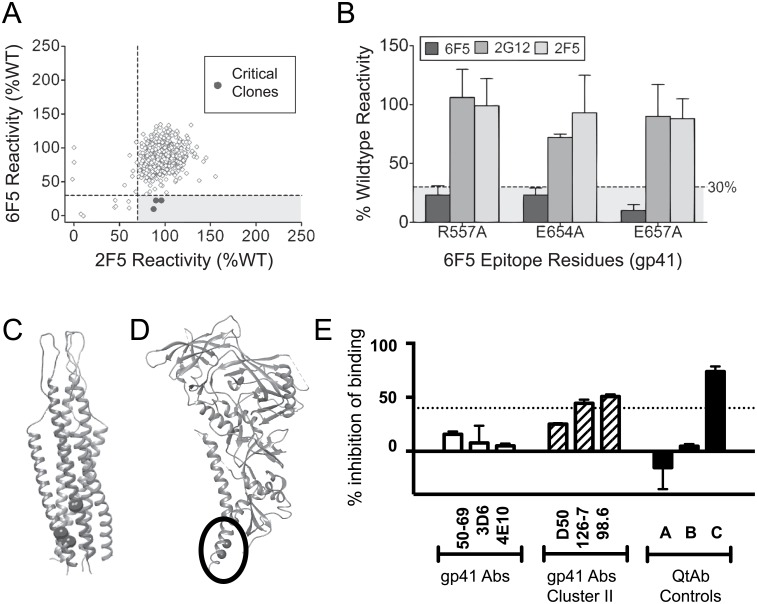
Identification of critical binding residues in the epitope for QtAb 6F5 by shotgun mutagenesis epitope mapping. (A) Binding relative to wild-type HIV gp160 reactivity is plotted for each mutant for the test QtAb 6F5, relative to the control mAb (2F5). Clones with reactivity <35% for the test mAb but with >70% reactivity for the control mAb were identified as critical for test mAb binding. (B) Alanine mutation of three individual residues significantly reduced 6F5 binding (dark grey bars), but did not impair binding of control Abs (2G12 or 2F5, shown in light grey or mid grey). Bars represent the mean and range of two replicates. (C) Structural visualization of the critical residues required for 6F5 binding, which compose the Ab epitope. Critical residues are shown in one chain of the gp41 post-fusion trimer (PDB 2EZQ) and (D) within the black circle on BG505 SOSIP.664 HIV-1 Env trimer (PDB 4ZMJ). (E) Competition-binding of biotinylated QtAb 6F5 against CG A mAb 2C6, CG B mAb 7B6, and gp41 mAbs, including gp41-specific mAb 50–69, Cluster-I-specific mAb 3D6, MPER-specific mAb 4E10 (open bars), and cluster II Abs D50, 98.6 and 126–7 (diagonal hatched bars). Competition causing >40% inhibition of binding of the BCA, approximately twice the background % of controls (over broken line), was considered significant and is shown with the control unlabeled CG C QtAb 7C6 (filled bar) as well as mAbs 98.6 and 126–7.

To identify critical binding residues within the HIV Env epitopes required for mAb binding, we next screened a comprehensive mutation library [16] comprising gp160 variants produced by shotgun mutagenesis, with each residue in the Env sequence individually mutated to alanine (alanine residues were mutated to serine). The library contained 678 gp160 mutants (99.9% coverage of targeted residues 30–708; numbering according to strain KNH1144, GenBank AAW72237). cDNAs encoding each member of the mutation library were used to transfect human HEK-293T cells in a 384-well array format (one clone per well) and evaluated in parallel for mAb reactivity to trimeric Env protein on the surface of cells. Expression was measured by immunofluorescent Ab staining and detection by flow cytometry with comparison to binding by control Ab binding (example comparisons are shown in Figs 3A and 4A).

The epitopes for CG A QtAb 2C6 and CG D QtAb 8B10 were not resolved, but the CG B and C QtAbs showed clear results. CG B QtAbs 5C2, 5F4, 7B6, 8F6 mapped to the immunodominant region 1 of gp41 known as Cluster I (residues 590–604), which comprises a loop between C598 and C604 (Fig 3, Table 2, S2 Table). We initially showed that the QtAbs did not compete with gp41 mAbs 2F5, 4E10 or 50–69, however, none of these mAbs specifically recognize the Cluster I epitope. MAb 3D6 is a non-neutralizing Ab that maps to the Cluster I epitope, as demonstrated by specific binding to the peptide CSGKLICTTAVPW (HXB2 gp160 reference amino acids 598–610) [21]. Notably, the nucleotide sequence encoding mAb 3D6 is nearly 97% identical to its inferred V_H_ and J_H_ germline genes encoding the heavy chain variable sequence [22], which starkly contrasts with the highly mutated QtAbs targeting this epitope that we isolated (82–85% of comparative heavy chain germline; S1 Table and [12]). In Ab competition-binding assays, we did not observe significant competition with mAb 3D6 or any of a selection of additional anti-gp41 Abs (Fig 3E). This lack of competition supports the idea that these new Abs target this epitope in a unique manner. Close review of the peptide mapping performed with mAb 7B6 (described above) showed that there was no specific binding to the five peptides corresponding to this region of amino acids 596–605. Overall homology of the extracellular portion of gp41, between group M consensus sequence and BaL, was 92.4% and the critical mapped residues were all homologous. The sequence of the peptides that contained the critical residues (NIH AIDS Reagent Products Cat # 9118–9122) overall were 93.3%, 100%, 93.3%, 93.3% and 86.7% homologous to that of the BaL strain, respectively. Of note, mAb 3D6 is known to bind linear epitopes, further supporting the differential targeting of this region [21].

**Table 2:** Disruption of mAb binding to HIV-1 gp160 by alanine replacement of amino acid residues.

Competition Group (CG)	mAb	HIV-1 gp160 mutations that disrupt binding	Region of HIV-1 gp160 containing epitope		
			gp41- Cluster I	gp41- Cluster II	Heptad Repeat 1
B	5C2	W596A, C598A, G600A, L602A, C604A	**X**		
	5F4	W596A, C598A, G600A, C604A	**X**		
	7B6	W596A, C598A, G600A, C604A	**X**		
	8F6	W596A, C598A, G600A, L602A, I603A, C604A	**X**		
C	4E4	N340A*, E657A		**X**	
	6F5	R557A, E654A, E657A		**X**	**X**
	6F11	E657A		**X**	
	7C6	E657A		**X**	

Underline indicates cysteines that are involved in disulfide bonds.

*Residue involved in a glycosylation site.

CG C QtAbs bound critical residues that mapped to the Cluster II region (residues 644–663) of gp41 (Fig 4, Table 2, S2 Table). Binding of each of the four CG C QtAbs (4E4, 6F5, 6F11, 7C6) depended on amino acid E657 within Cluster II. Cluster II is located adjacent to the membrane proximal region (MPER, residues 662–683), and is generally 100-fold less immunogenic than Cluster I [23]. Many of the Cluster II-specific mAbs only bind oligomeric forms of gp41 and are thought to predominantly recognize post-fusion forms of Env [24]. QtAb competition-binding against known cluster II-targeting mAbs gave varying results (Fig 4E). MAb D50 [25], which recognizes a linear epitope in the region, did not show significant competition. However, two Abs (designated 98.6 and 126.7, Fig 4E) that bind discontinuous epitopes on oligomeric gp41 with overlap in the Cluster II region, did show partial competition [26, 27].

There were notable significant findings outside of the Cluster II region as well. In addition to E657, resolved amino acids for mAb 4E4 included N340, a gp120 residue located within the V3 loop. This area of the V3 loop is moderately immunogenic, however binding in this assay is of uncertain significance (see note below in Discussion section). The more striking finding was that mAb 6F5 resolved another Cluster II residue, E654, as well as residue R557 that is far-removed in the primary sequence and located in the heptad repeat I region (residues 553–585) (Fig 4). Additional CG C Abs exhibited modest decreases in binding with these point mutations (see S2 Table: residue E654 (48–49%) and residue R557 (60–70%). Mapping of these residues on the structure of the SIV gp41, the first trimerized stalk structure resolved from a related retrovirus (PDB 2EZQ), placed these sites in close proximity, consistent with the formation of a discontinuous epitope (Fig 4C); however only E654 and E657 were resolved (Fig 4D, black thick circle) on the BG505 SOSIP.664 HIV-1 Env structure (PDB 4ZMJ). On review of the group M consensus peptide mapping data, even single peptides containing R557, E654 and E657 lacked specific binding to CG C Ab 7C6 (data not shown). The specific peptides (NIH AIDS Reagent Program Cat #9133 and Cat# 9111) containing critical residues of CG C showed 100% homology to the BaL strain originally used to clone these QtAbs and within the VLPs and trimers. Previous studies showed the extracellular domains of III B strain gp41 [12] has an overall homology of 92.4% to the BaL strain, and had complete conservation over long stretches of linear sequence (> 30 residues) that contain these critical residues (data not shown). The epitopes recognized by CG C QtAbs also likely include additional residues, but these amino acids could not be identified, either because they individually contributed only weakly to Ab binding or because they induced protein misfolding. These data reveal a structural epitope consisting of amino acids in Cluster II and the heptad repeat I region.

Abs to the Cluster I region have been shown previously to exhibit little preference for binding monomeric, dimeric or trimeric forms of gp140 antigen [26]. The CG B Abs described here, however, did show a preference for binding to VLPs over gp140 trimers (Table 1), generally with approximately 10-fold enhanced binding to VLPs. These mAbs did not bind monomeric gp41 [12] or individual peptides. CG C mAbs exhibited the most disparate intragroup binding kinetics (for example, mAb 4E4 in Fig 2) with mAbs 6F11 and 7C6 having essentially no preference for VLPs, and mAb 6F5 binding poorly to both gp140 trimers (Table 2). Despite this complexity, QtAbs 6F5 and 6F11 showed near identical results in competition-binding analysis (Fig 1). These data indicate that there is an inherent difference in epitope presentation by gp140 trimer constructs and trimers presented on VLPs. Since the epitopes described here are generally present on gp140 trimers, they are unlikely to represent solely post-fusion gp41 forms.

## Discussion

HIV-infected individuals make Abs to HIV Env soon after infection, but neutralizing responses in these individuals are often delayed. The immunodominant epitopes recognized by non-neutralizing Abs to gp41 early in infection are poorly understood. Here we determined the patterns of recognition of two groups of highly mutated naturally-occurring HIV-specific Abs that recognize two unique quaternary epitopes. We further defined a unique conformation-dependent quaternary epitope in the immunodominant Cluster-1 region of gp41 and characterized a unique discontinuous epitope not previously described.

These studies suggest that VLPs (and by implication, virions) display complex HIV Env antigens differently than typical trimeric gp140 forms of recombinant gp140. Despite the maintenance of the cleavage site, the VLPs used in this study already were known to retain surface gp120 [9], present broad neutralizing epitopes, and show typical exposure of epitopes after binding CD4, and also identified anti-gp120 B cells [10]. Recent studies suggest that many recombinant trimeric gp140s, such as the ones used here, do not fully recapitulate immunodominant quaternary epitopes found on infectious virions [28]. This finding is consistent with previously reviewed data from experiments in which labeled gp140 trimers constructed using the T4 foldon domain were used in a similar fashion to our use of labeled-VLPs to clone Ab-expressing genes from individual B cells. All of the cloned and expressed Abs in those studies bound to monomeric forms of Env protein [7]. Recent similar single B cell sorting studies using the BG505 SOSIP.664 gp140 trimer, theorized to present the native trimer, did facilitate isolation of neutralizing quaternary epitope-targeting Abs [29].

Clearly, native trimers may preserve immunogenic epitopes better than other forms of Env, but there is perhaps a false assumption made by many that all anti-HIV Abs that recognize quaternary epitopes are broadly neutralizing. Virions are known to shed gp120 and present gp41 ‘stumps’ that may misdirect the immune response [30–32]. These altered forms of Env may very well have quaternary epitopes associated with them. Some groups have suggested that any Ab that binds a quaternary epitope or recognizes the native trimeric structure of Env is neutralizing [33–35]. Serum neutralizing responses often do target quaternary and discontinuous epitopes on HIV virions [36, 37]. Certainly some potently neutralizing mAbs recognize quaternary epitopes such as PG9, PG16 [2], and the recently described mAbs 3BC176, 3BC315 [38] and 8066 [39, 40]. However, the data presented here suggests that potent neutralizing QtAbs may be rare and may be best isolated by large-scale screening. The study also raises the possibility that during HIV infection, the typical QtAbs that dominate the Ab response to complex Env epitopes are non-neutralizing.

Other recent reports regarding conformational epitopes are notable. MAb PGT122 recognizes a complex epitope encompassing V1, V3 and surrounding glycans [41]. Both the gp120 V3/V4 regions [42] and the V2 regions near the α4β7-binding site [43] were described as having conformational epitopes in an SIV study. MAb 3BC176 is a novel QtAb that was described recently with a CD4-inducible binding pattern and moderate depth and breadth of neutralization [38]. The 3BC176 Ab may be similar to CG A 2C6 QtAb, which exhibited limited neutralization of CD4-triggered virus [11].

It is not clear what forms of Env best represent the state of Env on virion particles in a physiologic milieu, although recent studies on the BG505 sequence are encouraging [44]. Natural virions may display gp120/gp41 monomers, gp120-depleted gp41 stumps, and other incomplete or noninfectious oligomeric forms, besides functional trimers, that may act as immune decoys [30–32]. These alternate viral protein structures also may misdirect the immune response toward non-neutralizing epitopes on gp120 and gp41 glycoproteins. It is thought that the majority of Abs directed against gp41 immunodominant Clusters I and II are weakly or non-neutralizing, although some can mediate Ab-dependent cellular cytotoxicity, or restrict HIV replication in macrophages and immature dendritic cells [45–47]. The immunodominant Cluster I loop should be inaccessible if gp120 is fully engaged with gp41 in a complete trimer, so many of the Abs targeting this epitope are thought to recognize post-fusion forms of Env or gp41 stumps [30]. Cluster I mutations have variable effects on HIV. Mutating sites 596 and 608 increases virion shedding of gp120, and mutating sites 605 and 609 enhances viral entry into cells, but mutations at 602 and 603 do not seem to change the association between gp41 to gp120 [48], and they do not affect HIV infectivity [49]. MAb 3D6, though not broadly neutralizing, can inhibit HIV-1 replication in Langerhans cells and interstitial dendritic cells [50]. MAb 3D6 has been mapped to the Cluster I loop [21], yet does not significantly inhibit Env binding of CG B QtAbs. This finding suggests that QtAbs recognize a variant form of the Cluster I loop, indicating this loop may be present in multiple conformations within the same assay. Differential binding to soluble trimers and VLPs (Table 1) further supports that the structural context of presentation of Env is particularly important for Cluster I epitopes. This observation is not surprising as this region may undergo complex dynamic changes as a ‘hinge’ during HIV fusion. One of the more extensive studies analyzing the Abs from B-cell clones that bind gp41 did find a small selection of potential conformationally targeted Abs that bound both the Cluster I region and MPER (Cluster IV) in gp41 [25].

Similarly, Cluster II-specific Abs are thought to be non-neutralizing due to the presumed targeting of a late-stage conformation of gp41. This conformation is thought to be only available once fusion is complete, or once gp120 has been ‘shed’, leaving decoy gp41 ‘stumps’ on the virion surface [51, 52]. Many Cluster II Abs only bind oligomeric forms of gp41 and are thought to predominantly recognize post-fusion forms of Env [24]. The QtAbs described here may be from clonal lineages that were initially responding to such structures. However, our data support the idea that forms of this Cluster II region may be more universally accessible. Specifically, we directly isolated these QtAbs from circulating patient B cells using membrane-bound, non-fused, VLPs, mapped them using cell surface expression of gp160, and these QtAbs bound *in vitro* to gp140 soluble trimers and to VLPs.

Residue E654 has been involved with other mAbs that bind to gp140 oligomers and not to peptides bearing linear gp41 sequences [53], a finding that is consistent with the data here. Although Cluster II Abs have been reported, such as mAbs 98.6 and 126–7, whose binding depends on oligomerization and approximation of heptad repeat I and heptad repeat II, this report is the first case of of which we are aware of specific resolution of R557 in an epitope. Notably, mAb 8066 binds heptad repeat I and is broadly neutralizing, however, this Ab epitope was mapped to amino acids H564, W571, K574, and Q575 [39, 40].

The CG C QtAbs described exhibited the greatest diversity in recognizing different forms of Env. Although initially characterized as separate clonal lineages [12], the similar sequence and shared epitope of these CG C QtAbs implies these Abs may share a clonal origin. Differences in binding patterns could reflect the effect of multiple Ab gene mutations that were selected over time. Particularly notable is MAb 6F5, which was the only QtAb for which alteration of the amino acid R557 abrogated binding. Within the epitope CG C, 6F5 also was the QtAb most sensitive to the change of antigen presentation from VLP to trimeric forms, and differentially recognized strain BaL versus SF162 (see Table 1). To our knowledge, Abs have not been specifically mapped to either this Cluster II residue (E654) or the heptad repeat I residue R557. Mab 4E4 also maps to N340, a glycosylation site residue in gp120 (24% reactivity when altered), and alteration of the residue T342 in its complementary glycosylation site caused only a moderate effect on binding (binding of about 76% of *wt*). Generally, this finding would argue against the site being an important part of the 4E4 epitope. When projected onto the structure recently reported for the strain BG505 SOSIP 664 trimer [41], this site is removed from residues R557 and S657 (data not shown). However, other conformational-dependent Abs, such as PGT151 [54] and 8ANC19 [55], are known to be affected by remote glycosylation sites.

The data shows that QtAbs can bind Env in a variety of trimeric constructions, even in some pre-fusion forms of Env. Env is thought to be particularly flexible on the membrane surface, and these Abs may only bind certain conformations of trimer [56]. Others have shown that even broadly neutralizing Abs targeting the same epitope sequentially interact and likely neutralize in structurally distinct ways [57]. The results from this study have direct relevance to current strategies for developing new experimental vaccines for HIV. Much of the current focus in the field is appropriately aimed at recapitulating Env antigens with conformational fidelity to the Env form found on infectious virions. While this objective is logical, our data suggest that it may be desirable to develop an engineered Env antigen that retains conserved neutralizing determinants but obscures immunodominant quaternary epitopes that frequently induce non-neutralizing Abs, like those shown in this manuscript. This approach is ongoing with the SOSIP trimer 664 from strain BG505 [44, 58]. Newer structure-based vaccine antigens designed using emerging computational methods might provide a way forward for development of optimized Env vaccines [59]. Further characterization of immunodominant epitopes that are undesirable in an optimal HIV immunogen should be of high interest to those engaged in rational vaccine design.

## Supporting Information

S1 Table Analysis of Ab variable genes encoding QtAbs analyzed functionally in this study. (DOCX)

S2 Table Identification of critical residues involved in mAb binding. (DOCX)

## References

[1] Burton DR, Stanfield RL, Wilson IA. Antibody vs. HIV in a clash of evolutionary titans. PNAS USA. 2005;102: 14943–8. 16219699 10.1073/pnas.0505126102 PMC1257708

[2] Walker LM, Phogat SK, Chan-Hui PY, Wagner D, Phung P, Goss JL, et al Broad and potent neutralizing antibodies from an African donor reveal a new HIV-1 vaccine target. Science. 2009;326: 285–9. 10.1126/science.1178746 19729618 PMC3335270

[3] Doria-Rose NA, Schramm CA, Gorman J, Moore PL, Bhiman JN, DeKosky BJ, et al Developmental pathway for potent V1V2-directed HIV-neutralizing antibodies. Nature. 2014;509: 55–62. 10.1038/nature13036 24590074 PMC4395007

[4] Mikell I, Sather DN, Kalams SA, Altfeld M, Alter G, Stamatatos L. Characteristics of the earliest cross-neutralizing antibody response to HIV-1. PLoS Path. 2011;7: e1001251. 10.1371/journal.ppat.1001251 PMC3020924 21249232

[5] Beddows S, Franti M, Dey AK, Kirschner M, Iyer SP, Fisch DC, et al A comparative immunogenicity study in rabbits of disulfide-stabilized, proteolytically cleaved, soluble trimeric human immunodeficiency virus type 1 gp140, trimeric cleavage-defective gp140 and monomeric gp120. Virology. 2007;360: 329–40. 17126869 10.1016/j.virol.2006.10.032

[6] VanCott TC, Bethke FR, Burke DS, Redfield RR, Birx DL. Lack of induction of antibodies specific for conserved, discontinuous epitopes of HIV-1 envelope glycoprotein by candidate AIDS vaccines. J Immunol. 1995;155: 4100–10. 7561123

[7] Scheid JF, Mouquet H, Feldhahn N, Seaman MS, Velinzon K, Pietzsch J, et al Broad diversity of neutralizing antibodies isolated from memory B cells in HIV-infected individuals. Nature. 2009;458: 636–40. 10.1038/nature07930 19287373

[8] Sundling C, Forsell MN, O'Dell S, Feng Y, Chakrabarti B, Rao SS, et al Soluble HIV-1 Env trimers in adjuvant elicit potent and diverse functional B cell responses in primates. J Exp Med. 2010;207: 2003–17. 10.1084/jem.20100025 20679401 PMC2931166

[9] Hammonds J, Chen X, Zhang X, Lee F, Spearman P. Advances in methods for the production, purification, and characterization of HIV-1 Gag-Env pseudovirion vaccines. Vaccine. 2007;25: 8036–48. 17936444 10.1016/j.vaccine.2007.09.016

[10] Hicar MD, Chen X, Briney B, Hammonds J, Wang JJ, Kalams S, et al Pseudovirion particles bearing native HIV envelope trimers facilitate a novel method for generating human neutralizing monoclonal antibodies against HIV. J Acquir Immune Defic Syndr. 2010;54: 223–35. 20531016 10.1097/QAI.0b013e3181dc98a3 PMC2930513

[11] Hicar MD, Kalams SA, Spearman PW, Crowe JE Jr., Emerging studies of human HIV-specific antibody repertoires. Vaccine. 2010;28 Suppl 2:B18–23. 10.1016/j.vaccine.2010.02.002 20510738 PMC2879399

[12] Hicar MD, Chen X, Kalams SA, Sojar H, Landucci G, Forthal DN, et al Low frequency of broadly neutralizing HIV antibodies during chronic infection even in quaternary epitope targeting antibodies containing large numbers of somatic mutations. Mol Immunol. 2015; 70:94–103. 10.1016/j.molimm.2015.12.002 26748387 PMC4762738

[13] Yang X, Farzan M, Wyatt R, Sodroski J. Characterization of stable, soluble trimers containing complete ectodomains of human immunodeficiency virus type 1 envelope glycoproteins. Journal of virology. 2000;74: 5716–25. 10823881 10.1128/jvi.74.12.5716-5725.2000 PMC112061

[14] Stamatatos L, Lim M, Cheng-Mayer C. Generation and structural analysis of soluble oligomeric gp140 envelope proteins derived from neutralization-resistant and neutralization-susceptible primary HIV type 1 isolates. AIDS Res Hum Retrov. 2000;16: 981–94. 10.1089/08892220050058407 10890360

[15] Sellhorn G, Caldwell Z, Mineart C, Stamatatos L. Improving the expression of recombinant soluble HIV Envelope glycoproteins using pseudo-stable transient transfection. Vaccine. 2009;28: 430–6. 10.1016/j.vaccine.2009.10.028 19857451

[16] Paes C, Ingalls J, Kampani K, Sulli C, Kakkar E, Murray M, et al Atomic-level mapping of antibody epitopes on a GPCR. J Am Chem Soc. 2009;131: 6952–4. 10.1021/ja900186n 19453194 PMC2943208

[17] Caffrey M, Cai M, Kaufman J, Stahl SJ, Wingfield PT, Covell DG, et al Three-dimensional solution structure of the 44 kDa ectodomain of SIV gp41. EMBO J. 1998;17: 4572–84. 9707417 10.1093/emboj/17.16.4572 PMC1170787

[18] Do Kwon Y, Pancera M, Acharya P, Georgiev IS, Crooks ET, Gorman J, et al Crystal structure, conformational fixation and entry-related interactions of mature ligand-free HIV-1 Env. Nat Struct Mol Biol. 2015;22: 522–31. 10.1038/nsmb.3051 26098315 PMC4706170

[19] Gorny MK, Stamatatos L, Volsky B, Revesz K, Williams C, Wang XH, et al Identification of a new quaternary neutralizing epitope on human immunodeficiency virus type 1 virus particles. J Virol. 2005;79: 5232–7. 10.1128/JVI.79.8.5232-5237.2005 15795308 PMC1069558

[20] Briggs WE. A new measure of cooperativity in protein-ligand binding. Biophys Chem. 1983;18: 67–71. 17005123 10.1016/0301-4622(83)80028-3

[21] Stigler RD, Ruker F, Katinger D, Elliott G, Hohne W, Henklein P, et al Interaction between a Fab fragment against gp41 of human immunodeficiency virus 1 and its peptide epitope: characterization using a peptide epitope library and molecular modeling. Protein Eng. 1995;8: 471–9. 8532669 10.1093/protein/8.5.471

[22] Kunert R, Ruker F, Katinger H. Molecular characterization of five neutralizing anti-HIV type 1 antibodies: identification of nonconventional D segments in the human monoclonal antibodies 2G12 and 2F5. AIDS Res Hum Retrov. 1998;14: 1115–28. 10.1089/aid.1998.14.1115 9737583

[23] Xu JY, Gorny MK, Palker T, Karwowska S, Zolla-Pazner S. Epitope mapping of two immunodominant domains of gp41, the transmembrane protein of human immunodeficiency virus type 1, using ten human monoclonal antibodies. J Virol. 1991;65: 4832–8. 1714520 10.1128/jvi.65.9.4832-4838.1991 PMC248941

[24] Gorny MK, Zolla-Pazner S. Recognition by human monoclonal antibodies of free and complexed peptides representing the prefusogenic and fusogenic forms of human immunodeficiency virus type 1 gp41. J Virol. 2000;74: 6186–92. 10846104 10.1128/jvi.74.13.6186-6192.2000 PMC112119

[25] Pietzsch J, Scheid JF, Mouquet H, Seaman MS, Broder CC, Nussenzweig MC. Anti-gp41 antibodies cloned from HIV-infected patients with broadly neutralizing serologic activity. J Virol. 2010;84: 5032–42. 10.1128/JVI.00154-10 20219932 PMC2863839

[26] Yuan W, Li X, Kasterka M, Gorny MK, Zolla-Pazner S, Sodroski J. Oligomer-specific conformations of the human immunodeficiency virus (HIV-1) gp41 envelope glycoprotein ectodomain recognized by human monoclonal antibodies. AIDS Res Hum Retrov. 2009;25: 319–28. 10.1089/aid.2008.0213 PMC2853836 19292593

[27] Vincent N, Malvoisin E. Ability of antibodies specific to the HIV-1 envelope glycoprotein to block the fusion inhibitor T20 in a cell-cell fusion assay. Immunobiology. 2012;217: 943–50. 22387075 10.1016/j.imbio.2012.01.007

[28] Ringe RP, Sanders RW, Yasmeen A, Kim HJ, Lee JH, Cupo A, et al Cleavage strongly influences whether soluble HIV-1 envelope glycoprotein trimers adopt a native-like conformation. PNAS USA. 2013;110: 18256–61. 10.1073/pnas.1314351110 24145402 PMC3831437

[29] Sok D, van Gils MJ, Pauthner M, Julien JP, Saye-Francisco KL, Hsueh J, et al Recombinant HIV envelope trimer selects for quaternary-dependent antibodies targeting the trimer apex. PNAS USA 2014;111: 17624–9. 10.1073/pnas.1415789111 25422458 PMC4267403

[30] Crooks ET, Moore PL, Franti M, Cayanan CS, Zhu P, Jiang P, et al A comparative immunogenicity study of HIV-1 virus-like particles bearing various forms of envelope proteins, particles bearing no envelope and soluble monomeric gp120. Virology. 2007;366: 245–62. 17580087 10.1016/j.virol.2007.04.033 PMC2080857

[31] Lin G, Nara PL. Designing immunogens to elicit broadly neutralizing antibodies to the HIV-1 envelope glycoprotein. Curr HIV Res. 2007;5: 514–41. 18045109 10.2174/157016207782418489

[32] Moore PL, Crooks ET, Porter L, Zhu P, Cayanan CS, Grise H, et al Nature of nonfunctional envelope proteins on the surface of human immunodeficiency virus type 1. J Virol. 2006;80: 2515–28. 16474158 10.1128/JVI.80.5.2515-2528.2006 PMC1395414

[33] Fouts TR, Binley JM, Trkola A, Robinson JE, Moore JP. Neutralization of the human immunodeficiency virus type 1 primary isolate JR-FL by human monoclonal antibodies correlates with antibody binding to the oligomeric form of the envelope glycoprotein complex. J Virol. 1997;71: 2779–85. 9060632 10.1128/jvi.71.4.2779-2785.1997 PMC191401

[34] Sattentau QJ, Moore JP. Human immunodeficiency virus type 1 neutralization is determined by epitope exposure on the gp120 oligomer. J Exp Med. 1995;182: 185–96. 7540648 10.1084/jem.182.1.185 PMC2192089

[35] Parren PW, Mondor I, Naniche D, Ditzel HJ, Klasse PJ, Burton DR, et al Neutralization of human immunodeficiency virus type 1 by antibody to gp120 is determined primarily by occupancy of sites on the virion irrespective of epitope specificity. J Virol. 1998;72: 3512–9. 9557629 10.1128/jvi.72.5.3512-3519.1998 PMC109569

[36] Moore JP, Ho DD. Antibodies to discontinuous or conformationally sensitive epitopes on the gp120 glycoprotein of human immunodeficiency virus type 1 are highly prevalent in sera of infected humans. J Virol. 1993;67: 863–75. 7678308 10.1128/jvi.67.2.863-875.1993 PMC237440

[37] Steimer KS, Haigwood NL. Importance of conformation on the neutralizing antibody response to HIV-1 gp120. Biotechnol Ther. 1991;2: 63–89. 1726963

[38] Klein F, Gaebler C, Mouquet H, Sather DN, Lehmann C, Scheid JF, et al Broad neutralization by a combination of antibodies recognizing the CD4 binding site and a new conformational epitope on the HIV-1 envelope protein. J Exp Med. 2012;209: 1469–79. 10.1084/jem.20120423 22826297 PMC3409500

[39] Louis JM, Aniana A, Lohith K, Sayer JM, Roche J, Bewley CA, et al Binding of HIV-1 gp41-directed neutralizing and non-neutralizing fragment antibody binding domain (Fab) and single chain variable fragment (ScFv) antibodies to the ectodomain of gp41 in the pre-hairpin and six-helix bundle conformations. PLoS One. 2014;9: e104683 10.1371/journal.pone.0104683 25105806 PMC4126735

[40] Gustchina E, Li M, Ghirlando R, Schuck P, Louis JM, Pierson J, et al Complexes of neutralizing and non-neutralizing affinity matured Fabs with a mimetic of the internal trimeric coiled-coil of HIV-1 gp41. PLoS One. 2013;8: e78187 10.1371/journal.pone.0078187 24244293 PMC3820714

[41] Julien JP, Cupo A, Sok D, Stanfield RL, Lyumkis D, Deller MC, et al Crystal structure of a soluble cleaved HIV-1 envelope trimer. Science. 2013;342: 1477–83. 10.1126/science.1245625 24179159 PMC3886632

[42] Kuwata T, Takaki K, Yoshimura K, Enomoto I, Wu F, Ourmanov I, et al Conformational epitope consisting of the V3 and V4 loops as a target for potent and broad neutralization of simian immunodeficiency viruses. J Virol. 2013;87: 5424–36. 10.1128/JVI.00201-13 23468483 PMC3648159

[43] Mayr LM, Cohen S, Spurrier B, Kong XP, Zolla-Pazner S. Epitope mapping of conformational V2-specific anti-HIV human monoclonal antibodies reveals an immunodominant site in V2. PLoS One. 2013;8: e70859 10.1371/journal.pone.0070859 23923028 PMC3726596

[44] Sanders RW, Derking R, Cupo A, Julien JP, Yasmeen A, de Val N, et al A next-generation cleaved, soluble HIV-1 Env Trimer, BG505 SOSIP.664 gp140, expresses multiple epitopes for broadly neutralizing but not non-neutralizing antibodies. PLoS Path. 2013;9: e1003618. 10.1371/journal.ppat.1003618 PMC3777863 24068931

[45] Holl V, Peressin M, Decoville T, Schmidt S, Zolla-Pazner S, Aubertin AM, et al Nonneutralizing antibodies are able to inhibit human immunodeficiency virus type 1 replication in macrophages and immature dendritic cells. J Virol. 2006;80: 6177–81. 16731957 10.1128/JVI.02625-05 PMC1472578

[46] Tomaras GD, Yates NL, Liu P, Qin L, Fouda GG, Chavez LL, et al Initial B-cell responses to transmitted human immunodeficiency virus type 1: virion-binding immunoglobulin M (IgM) and IgG antibodies followed by plasma anti-gp41 antibodies with ineffective control of initial viremia. J Virol. 2008;82: 12449–63. 10.1128/JVI.01708-08 18842730 PMC2593361

[47] Tyler DS, Stanley SD, Zolla-Pazner S, Gorny MK, Shadduck PP, Langlois AJ, et al Identification of sites within gp41 that serve as targets for antibody-dependent cellular cytotoxicity by using human monoclonal antibodies. J Immunol. 1990;145: 3276–82. 1700004

[48] Jacobs A, Sen J, Rong L, Caffrey M. Alanine scanning mutants of the HIV gp41 loop. J Biol Chem. 2005;280: 27284–8. 15917239 10.1074/jbc.M414411200

[49] Liao HX, Chen X, Munshaw S, Zhang R, Marshall DJ, Vandergrift N, et al Initial antibodies binding to HIV-1 gp41 in acutely infected subjects are polyreactive and highly mutated. J Exp Med. 2011;208: 2237–49. 10.1084/jem.20110363 21987658 PMC3201211

[50] Peressin M, Holl V, Schmidt S, Decoville T, Mirisky D, Lederle A, et al HIV-1 replication in Langerhans and interstitial dendritic cells is inhibited by neutralizing and Fc-mediated inhibitory antibodies. J Virol. 2011;85: 1077–85. 10.1128/JVI.01619-10 21084491 PMC3020030

[51] Frey G, Chen J, Rits-Volloch S, Freeman MM, Zolla-Pazner S, Chen B. Distinct conformational states of HIV-1 gp41 are recognized by neutralizing and non-neutralizing antibodies. Nat Struct Mol Biol. 2010;17: 1486–91. 10.1038/nsmb.1950 21076402 PMC2997185

[52] Poignard P, Moulard M, Golez E, Vivona V, Franti M, Venturini S, et al Heterogeneity of envelope molecules expressed on primary human immunodeficiency virus type 1 particles as probed by the binding of neutralizing and nonneutralizing antibodies. J Virol. 2003;77: 353–65. 12477840 10.1128/JVI.77.1.353-365.2003 PMC140593

[53] Dennison SM, Anasti K, Scearce RM, Sutherland L, Parks R, Xia SM, et al Nonneutralizing HIV-1 gp41 envelope cluster II human monoclonal antibodies show polyreactivity for binding to phospholipids and protein autoantigens. J Virol. 2011;85: 1340–7. 10.1128/JVI.01680-10 21106741 PMC3020517

[54] Blattner C, Lee JH, Sliepen K, Derking R, Falkowska E, de la Pena AT, et al Structural delineation of a quaternary, cleavage-dependent epitope at the gp41-gp120 interface on intact HIV-1 Env trimers. Immunity. 2014;40: 669–80. 10.1016/j.immuni.2014.04.008 24768348 PMC4057017

[55] Scharf L, Scheid JF, Lee JH, West AP Jr., Chen C, Gao H, et al Antibody 8ANC195 reveals a site of broad vulnerability on the HIV-1 envelope spike. Cell Reports. 2014;7: 785–95. 10.1016/j.celrep.2014.04.001 24767986 PMC4109818

[56] Pancera M, Zhou T, Druz A, Georgiev IS, Soto C, Gorman J, et al Structure and immune recognition of trimeric pre-fusion HIV-1 Env. Nature. 2014;514: 455–61. 10.1038/nature13808 25296255 PMC4348022

[57] Tran EE, Borgnia MJ, Kuybeda O, Schauder DM, Bartesaghi A, Frank GA, et al Structural mechanism of trimeric HIV-1 envelope glycoprotein activation. PLoS Path. 2012;8: e1002797. 10.1371/journal.ppat.1002797 PMC3395603 22807678

[58] Hoffenberg S, Powell R, Carpov A, Wagner D, Wilson A, Kosakovsky Pond S, et al Identification of an HIV-1 clade A envelope that exhibits broad antigenicity and neutralization sensitivity and elicits antibodies targeting three distinct epitopes. JVirol. 2013;87: 5372–83. 23468492 10.1128/JVI.02827-12 PMC3648150

[59] Schief WR, Ban YE, Stamatatos L. Challenges for structure-based HIV vaccine design. Curr Opin HIV AIDS. 2009;4: 431–40. 20048708 10.1097/COH.0b013e32832e6184

