# Supplementary material for: Human Antibodies that Recognize Novel Immunodominant Quaternary Epitopes on the HIV-1 Env Protein
Source: PLoS One. 2016 Jul 13;11(7):e0158861. doi: 10.1371/journal.pone.0158861 (PMC4943599; doi:10.1371/journal.pone.0158861)
Supplement: S1 Table — (DOCX) [file pone.0158861.s001.docx]

| **S1 Table. Epitope groups and variable gene characteristics of the QtAbs analyzed in this study** | | | | | | | | | | | | |
| --- | --- | --- | --- | --- | --- | --- | --- | --- | --- | --- | --- | --- |
| **Antibody** | | **Heavy chain variable gene sequence** | | | | | | **Light chain variable gene sequence** | | | | |
| **Clone^1^** | **Competition Group** | **V_H_ gene** | **nt % homo-logy**^2^ | **D_H_**  **gene** | **J_H_ gene** | **HCDR3**  **amino acids (aa)** | **CDR3 length (aa)** | **V_K_**  **gene** | **nt % homo-logy**^2^ | **J_K_ gene** | **LCDR3**  **amino acids (aa)** | **CDR3 length (aa)** |
| Q3-2C6 | A | 1-69 | 86.4 | 4-17 | 6 | CARGMIHADYRSDPFYHYYMDVW | 21 | 1-27 | 94.9 | 3 | CQSYNSAHFTF | 9 |
| Q4-5F4 | B | 1-69 | 83.0 | 3-10 | 5 | CANSRLYYEGPLLTGVGYFDPW | 20 | 1-5 | 93.4 | 1 | CQQYENYPRTF | 9 |
| Q5-5C2 |  | 1-69 | 81.8 | 3-16 | 1 | CAGSRMYYEGGLLTGVGYFDPW | 20 | 1-5 | 90.2 | 1 | CQQYENYPRTF | 9 |
| Q5-8F6 |  | 1-69 | 81.8 | 3-16 | 1 | CAGSRMYYEGGLLTGVGYFDPW | 20 | 1-5 | 89.8 | 1 | CQQYENYPRTF | 9 |
| Q6-7B6 |  | 1-69 | 85.2 | 3-10 | 5 | CSNSRLYYEGGLLTGVGWFGPW | 20 | 1-5 | 93.0 | 1 | CQQYHDYPRTF | 9 |
| Q7-6F11 | C | 1-2 | 83.0 | 6-6 | 4 | CGRTSIASRHLDSW | 12 | 1-39 | 94.5 | 2 | CQETYSVPPWTF | 10 |
| Q7-7C6 |  | 1-2 | 79.6 | 6-6 | 4 | CGRTSIASRHLDSW | 12 | 1-39 | 86.7 | 2 | CQETYSVPPWTF | 10 |
| Q11-4E4^3^ |  | 1-2 | 87.5 | 2-15 | 3 | CARGEIAGRRLDLW | 12 | nd | nd | nd | nd | nd |
| Q13-6F5 |  | 1-2 | 83.0 | 1-26 | 4 | CARVQMAGRDADLW | 12 | 1-39 | 90.6 | 1 | CQQTYASPPWTF | 10 |
| Q14-8B10 | D | 4-59 | 91.6 | 4-23 | 4 | CARVRKAMSTVAFDSW | 14 | 1D-12 | 93.4 | 5 | CQLTNTFLGITF | 10 |

^1^Q group designation was assigned previously by nucleotide sequence homology and show these ten Abs form eight sequence similarity groups; see reference 12.

^2^V_H_ or V_L_ gene percent homology to closest germline V_H_ or V_L_ gene segment, as predicted by IMGT.

^3^Antibody clone Q11-4E4 represents a chimeric antibody of the 76-Q11-4E4 heavy chain co-expressed with the 76-Q13-6F5 kappa chain.
