# Supplementary material for: Human Antibodies that Recognize Novel Immunodominant Quaternary Epitopes on the HIV-1 Env Protein
Source: PLoS One. 2016 Jul 13;11(7):e0158861. doi: 10.1371/journal.pone.0158861 (PMC4943599; doi:10.1371/journal.pone.0158861)
Supplement: S2 Table — (DOCX) [file pone.0158861.s002.docx]

| **S2 Table. Identification of critical residues involved in mAb binding.** | | | | | | | | | | |
| --- | --- | --- | --- | --- | --- | --- | --- | --- | --- | --- |
| **Cluster and Group**  **(gp41 region)** | **mAb** | **Antibody reactivity to critical residues [Mean of percentage of binding to wild-type gp160 (range)] in immunofluorescence binding assay, using cell surface display of gp160** | | | | | | | | |
|  |  | **R557** | **W596** | **C598^1^** | **G600** | **L602** | **I603** | **C604^1^** | **E654** | **E657** |
| Cluster I  (Group B) | 5C2 | 70 (3) | **8 (3)^2^** | **-3 (5)** | **7 (1)** | **21 (4)** | 45 (4) | **-6 (3)** | 75 (7) | 65 (38) |
|  | 5F4 | 73 (46) | **20 (2)** | **20 (5)** | **37 (7)** | 73 (3) | 59 (16) | **18 (8)** | 63 (21) | 68 (7) |
|  | 7B6 | 82 (26) | **-1 (14)** | **3 (16)** | **32 (1)** | 39 (60) | 58 (16) | **3 (12)** | 120 (19) | 90 (43) |
|  | 8F6 | 96 (60) | **12 (7)** | **2 (9)** | **22 (3)** | **32 (27)** | **35 (2)** | **12 (25)** | 97 (6) | 65 (28) |
| Cluster II  (Group B) | 4E4 | 60 (1) **^2^** | 78 (8) | 64 (12) | 51 (13) | 62 (3) | 94 (3) | 82 (14) | 48 (10) | **15 (1) ^3^** |
|  | 6F5 | **23 (8)** | 97 (53) | 85 (38) | 86 (22) | 96 (24) | 103 (45) | 81 (39) | **23 (6)** | **10 (5)** |
|  | 6F11 | 63 (38) | 96 (37) | 79 (15) | 86 (43) | 86 (19) | 91 (14) | 89 (19) | 49 (7) | **26 (21)** |
|  | 7C6 | 70 (38) | 66 (64) | 78 (15) | 77 (26) | 92 (45) | 89 (27) | 77 (24) | 48 (10) | **11 (6)** |
| Controls | 2G12 | 106 (24) | 73 (5) | 77 (19) | 103 (38) | 106 (8) | 72 (11) | 84 (7) | 72 (3) | 90 (27) |
|  | 2F5 | 99 (23) | 90 (18) | 107 (11) | 86 (25) | 101 (35) | 77 (23) | 75 (6) | 93 (32) | 88 (17) |

**^1^**Cysteine residues C598 and C604 form a disulfide bond.

^2^MAb reactivities for each alanine scan mutant are expressed as percent of wild-type with ranges (half of the maximum minus minimum values) in parentheses.

**^3^**Values for residues involved in mAb binding are highlighted in **bold**.
